# ‘Trifecta’ outcomes of robot-assisted partial nephrectomy: Results of the ‘low volume’ surgeon

**DOI:** 10.1590/S1677-5538.IBJU.2019.0396

**Published:** 2020-09-02

**Authors:** Cem Basatac, Haluk Akpinar

**Affiliations:** 1 Istanbul Bilim University Department of Urology Istanbul Turkey Department’s Istanbul Bilim University, Department of Urology, Istanbul, Turkey.

**Keywords:** Nephrectomy, Robotic Surgical Procedures, Surgeons

## Abstract

**Objective:**

There is limited data regarding surgeon volume and partial nephrectomy outcomes. The aim of this study is to report trifecta outcomes of robot-assisted partial nephrectomy (RAPN) performed by the low volume surgeon.

**Materials and Methods:**

Thirty-nine patients with clinical T1-2 renal tumors who underwent RAPN between 2012 and 2018 were included in this study. Trifecta was defined as negative surgical margins, warm ischemia time ≤20 minutes, and no operative complications. Patient demographics, R.E.N.A.L. nephrometry score, operation time, estimated blood loss, warm ischemia time, length of hospital stay, renal functions, and oncological outcomes were analyzed retrospectively. Complications were graded based on the modified Clavien-Dindo classification system.

**Results:**

The median R.E.N.A.L. nephrometry score was 6 (4-10). RAPN was successfully performed in all but one patient. The median operation time was 180 (90-240) minutes. Warm ischemia was performed only by segmental renal artery control in 35 and, by main renal artery control in three patients. The off-clamp technique was used in two patients. The median warm ischemia time was 16 (0-31) minutes. Seven patients had a warm ischemia time of longer than 20 minutes. Three patients had postoperative complications. The surgical margin was positive in one patient. As a result, the trifecta was achieved in 30 of the 39 patients (77%).

**Conclusion:**

RAPN is a safe and effective minimally invasive alternative in the treatment of renal masses. The present study suggests that reasonable trifecta rates can be achieved even by low volume surgeons.

## INTRODUCTION

The incidence of renal tumors has increased worldwide over the last two decades due to widespread use of cross-sectional imaging techniques ( 1 ). Nowadays, nephron-sparing surgery (NSS) remains the preferred approach for the treatment of T1a, and when technically feasible, in T1b renal tumors since it provides superior functional outcomes and less cardiovascular events when compared to radical nephrectomy ( 2 ). Among the NSSs, robot-assisted partial nephrectomy (RAPN) was introduced as a minimally invasive alternative to open and laparoscopic partial nephrectomy, with the numerous advantages of new technologies and has become increasingly preferred even in the more complex renal tumors ( 3 ). Most recently, a new concept called ‘Trifecta’ has been suggested to evaluate the success of RAPN. It was first reported by Hung et al. and defined as the simultaneous achievement of all three goals of RAPN including negative surgical margins, no operative complications, and renal functional preservation ( 4 ). Due to its technical complexity, it is likely that RAPN outcomes can be affected by provider volume. As has been shown in prior studies, higher surgeon volume was associated with better operative outcomes for radical prostatectomy and cystectomy; however, there are very few publications concerning the impact of surgeon volume on the RAPN outcomes ( 5 , 6 ). As a result, the aim of the present study was to evaluate trifecta outcomes of a low volume surgeon’s RAPN series and thus helping to fill a perceived gap in the literature, about the volume-outcome relationship of the RAPN.

## MATERIALS AND METHODS

The present study was approved by the Institutional Ethics Committee of Istanbul Bilim University, Faculty of Medicine (Approval number: 26.02.2019/2019-05-01). Written informed consent was obtained from all the patients. Robot-assisted partial nephrectomy was performed in 44 consecutive patients between March 2012 and December 2018. Of these patients, four were excluded due to radiological evidence of locally advanced disease and incomplete records. In addition, RAPN was successfully performed in all but one patient. Another patient in whom management was converted to total nephrectomy after two consecutive positive frozen section reports was also excluded from the study. The remaining 39 patients with renal tumors who underwent RAPN were included in this study. Among these, twenty-five were clinically staged as T1a, twelve as T1b and one was staged as T2. Surgeon volume was described as the number of procedures performed within a year by a single surgeon, regardless of the hospital volume. In the present study, the console surgeon was defined as a low volume surgeon, since 6.2 RAPN procedures were performed annually by him in several different hospitals during the study period. Although he was defined as “a low volume surgeon” for RAPN, the console surgeon of this cohort has been performing robotic surgeries regularly for fourteen years.

Preoperative evaluation was performed by medical history, physical examination, blood biochemistry, urinalysis and chest X-ray. Contrast-enhanced computed tomography (CT) with 3-D modification was used in all patients in order to delineate tumor characteristics and renal vascular anatomy. Pathological staging was performed according to the 2009 IUCC/American Joint Committee on Cancer tumor-node-metastasis staging system ( 7 ). All tumors were categorized according to the R.E.N.A.L. nephrometry score ( 8 ). Patient demographics, operation time, estimated blood loss (EBL), warm ischemia time (WIT), length of hospital stay, pre- and postoperative renal functions and oncological outcomes were prospectively registered in our database and analyzed retrospectively. Estimated glomerular filtration rates (eGFR) were calculated with the Modification of Diet and Renal Disease formula ( 9 ). Preoperative and postoperative 1^st^ month follow-up creatinine and eGFR levels were compared. All complications within 30 days after RAPN were graded based on the modified Clavien-Dindo classification system ( 10 ). Positive surgical margin was defined as the presence of tumor cells on the inked surface of the resected specimen on the pathology report. Trifecta was described as negative surgical margins, WIT less than 20 minutes, and no peri- and postoperative complications. During follow-up, all patients received a comprehensive metabolic panel every three months for the first two years and once a year subsequently. An abdominal CT and chest radiography were performed at the 3^rd^month. When necessary, abdominal ultrasonography or CT and chest radiography were performed by the 6^th^ month, and yearly thereafter.

### Surgical Technique

All operations were performed using the da Vinci SI robotic surgical system® (Intuitive Surgical, Inc., Sunnyvale, CA). A five-port transperitoneal approach was used for left-sided tumors. An additional 5mm port was used for liver retraction, for right-sided tumors. Following endotracheal intubation under general anesthesia, a ureteral catheter was placed in patients whose tumor was too close to the collecting system. The patient was then placed at a 60° modified flank position. Pneumoperitoneum was achieved with a Veress needle at Palmer’s point ( 11 ) for left renal tumors and 2 cm cranial from the midpoint between the umbilicus and anterior superior iliac spine for right renal tumors. The colon was reflected medially. The main renal vein, renal artery, and targeted segmental arteries were separately dissected and encircled by vascular loops in all cases. The renal capsule was scored using monopolar shears. Two 15 cm long 3-0 polyglyconate barbed sutures on a 26 mm ½ circle needle were placed in the abdominal cavity for renal parenchymal repair. Metal and plastic bulldog clamps of different sizes (Scanlan International, St. Paul, MN) were used in order to control segmental branches of the main renal artery supplying the tumor, depending on the size of the renal arterial branch. Cold excision of the tumor was performed with robotic hot shears. It was also necessary to clamp additional segmental arteries when arterial bleeding from tumor bed occurred. The pelvicalyceal system was repaired by uninterrupted 4-0 polyglactin suture, if opened up. The tumor bed was then sutured continuously with two preplaced barbed sutures. Subsequently, bulldog clamps were released (early unclamping). In case of pulsatile arterial bleeding, vessels were controlled in a figure of eight fashion by using 4-0 polyglactin suture. The renal parenchyma was further approximated using 0-0 polyglactin sutures on a CT-1 needle with the sliding-clip renorrhaphy technique ( 12 ). A Jackson-Pratt drain was placed in all patients.

### Statistical Analysis

All statistical analyses were performed using the SPSS Statistics version 24 (IBM, Armonk, NY, USA) software. The sample mean was used to determine the average of collected data as quantitative variables met the normal distribution according to the Kolmogorov-Smirnov test, otherwise, sample median was used. The paired-sample t-test was used to compare pre- and post-treatment differences within the groups. The confidence interval was taken 95%, and a P <0.05 was considered statistically significant.

## RESULTS

Demographics and operative outcomes of patients are listed in Table-1 . The median age was 57 (32-74) years. Median R.E.N.A.L nephrometry score and tumor size were 6 ( 4 - 10 ) and 38 (12-80) millimeters, respectively. No conversions were required. The median operation time was 180 (90-240) minutes. Warm ischemia was performed only through selective segmental renal artery control in 35, and by main renal artery control in two patients. The off-clamp technique was used in only two patients. The early unclamping technique was also used to limit warm ischemia time in all but three patients. Median WIT was 16 (0-31) minutes. Estimated blood loss was 200 (10-700) milliliters. The median drain removal time and length of hospital stay were 2 ( 1 - 5 ) and 3 ( 2 - 8 ) days, respectively. The mean decline in hematocrit was 4% (±2.12), and this was found to be statistically significant (p <0.01). Mean pre- and post-operative eGFR values (mL/min/1.73m^2^) were 89 (±23) and 85 (±24), respectively. The mean reduction in eGFR at one month after surgery was not statistically significant (p=0.18).

**Table 1:** Demographics and operative outcomes of patients.

Number of patients	39
Age, years	57 (32-74)
BMI	29 (26-41)
Tumor size, mm	38 (12-80)
R.E.N.A.L. Nephrometry score	6 (4-10)
Operation time, min	180 (90-240)
Warm ischemia time, min	16 (0-31)
Estimated blood loss, mL	200 (10-700)
Drain removal time, days	2 (1-5)
Length of stay, days	3 (2-8)

Data indicated as median (interquartile range), **BMI** = Body mass index

All the trifecta failure patients in our series are listed in Table-2 . Four complications recorded as Clavien grade 2 and 3-b were observed in three patients. These included two postoperative bleeding requiring blood transfusions, one pneumothorax requiring a chest tube, and one urinoma requiring percutaneous drainage. Final pathological examination of 39 resected tumors revealed 32 malignant tumors (82%) and seven benign tumors (18%). The surgical margin was found to be positive in one patient. However, no tumor recurrence was reported during a median follow-up of 44 (12-92) months. Warm ischemia time was found to be longer than 20 minutes in seven patients. Although the segmental renal artery clamping technique was used in all, these seven patients were not assigned as trifecta achieved. As a result, trifecta was achieved in 77% of the patients (30/39).

**Table 2:** Trifecta failure patients.

Case no.	Year of study		Age	Gender	RENAL score	Length of stay	Vascular control	WIT (Min)	Surgical margin	Clavien complication
3		1	58	M	8	2	SAC	25	-	0
9		3	74	M	6	5	SAC	22	-	3-b^a^
10		3	74	M	9	3	SAC	23	-	0
16		4	73	M	10	4	SAC	28	-	0
20		4	58	F	6	5	SAC	16	-	2-a and 3-b^b^
25		5	56	M	8	4	SAC	31	+	0
31		6	41	M	8	3	SAC	27	-	0
33		6	62	M	10	3	SAC	24	-	0
35		6	60	M	6	3	SAC	11	-	2-a^c^

**WIT** = Warm ischemia time, **SAC** =Segmental renal artery clamping.

^**a**^ pneumothorax treated by a chest tube drainage.

^**b**^ postoperative bleeding necessitating blood transfusion and urinoma treated by the percutaneous drainage.

^**c**^ postoperative bleeding necessitating blood transfusion.

## DISCUSSION

Robot-assisted partial nephrectomy is being increasingly used in the treatment of renal tumors with a number of reports describing favorable outcomes, when compared with laparoscopic and open partial nephrectomy ( 13 ). Robotic surgery provides significant advantages to the surgeon, including three-dimensional visualization, increased magnification and better depth perception which make tumor excision easy even in posteriorly located tumors. Published reports support that RAPN is associated with more favorable operative and functional outcomes, over open and laparoscopic partial nephrectomy ( 3 ). When compared with LPN, the main advantages of RAPN seems to be a shorter learning curve and reduced warm ischemia time, providing equivalent oncologic results as LPN ( 14 ). With increasing experience in the surgical technique, larger and more complex renal tumors have also been treated by RAPN with satisfactory oncologic and operative outcomes ( 15 ).

With the increasing popularity of RAPN among urologists, a surgical quality assessment score called “Trifecta” has emerged to determine the surgical success of the procedure. The largest RAPN series have reported their trifecta results as ranging from 32% to 86% ( 16 , 17 ). Zargar et al. compared trifecta outcomes of 2393 consecutive cases of robotic and laparoscopic partial nephrectomy performed by high-volume surgeons from five centers. It was reported that the RAPN group had superior outcomes in terms of WIT, overall complications and positive surgical margin rates, and they concluded that RAPN had significantly better trifecta outcomes when compared to LPN ( 17 ). In addition, trifecta outcomes have also been reported for RAPN in patients with T1b renal tumors ranging from 43% to 80% ( 16 - 19 ). These reports suggest that optimal trifecta outcomes may be achieved by RAPN even in patients with T1b renal tumors.

Trifecta for RAPN first defined by Hung et al. focuses on oncologic outcomes, renal functional preservation, and safety profile of the procedure ( 4 ). In the present study, cancer control was determined by the negative surgical margin on the final pathology report. The warm ischemia time was used to determine renal functional preservation and less than 20 minutes was described as the cutoff for achieving trifecta. The safety profile of the procedure was also assessed with the absence of peri- and postoperative complications. In the present low volume surgeon’s series, trifecta was achieved in 30/39 (77%) of the patients and this rate is considered as similar to previously reported series for RAPN by high volume surgeons depicted above ( 16 - 19 ).

Although conflicting reports exist relatied to the impact of surgeon volume on the outcomes of RAPN, higher surgeon volume seems to be associated with better postoperative outcomes ( 20 ). Several studies have shown that patients treated by high volume surgeons are more likely to have favorable operative and postoperative outcomes after RAPN ( 21 ). These findings were supported by the Khandwala et al. in a large population-based study. They analyzed the impact of surgeon volume on the perioperative outcomes of RAPN. The surgeons were divided based on the annual RAPN numbers and defined as very low, ≤2 cases/year, low, 3-4 cases/year; intermediate, 5-7 cases/year, high, 8-13 cases/year, and, very high, ≥14 cases/year. It was concluded that very high volume surgeons tend to have less major surgical complications, shorter operation time and fewer length of hospital stay according to results of this study ( 22 ). In a similar study, Peyronnet et al. also divided surgeons into four groups and compared the results. Low volume surgeons were defined as those who performed under seven RAPN/year, whereas very high volume surgeons were those who performed over 30 RAPN/year. The primary endpoint was trifecta achievement in that study. Although the trifecta achievement rate increased with surgeon volume, it was not statistically significant in a multivariate analysis adjusting both hospital and surgeon volume. It was finally stated by the authors that hospital volume had a greater impact on perioperative outcomes than surgeon volume ( 23 ).

In the present study, nearly 7 RAPN/year were performed by a single surgeon and trifecta was achieved in 77% of these patients. Although surgeon volume itself is considered as one of the important factors for partial nephrectomy outcomes as mentioned by the Dagenais et al., other measured and unmeasured surgeon factors may account for 18-100% of variability of peri- and postoperative variables such as years in practice, surgical approach, fellowship and modular training, etc. Results of the current study are also consistent with the one reported above ( 24 ).

The acceptable trifecta rates of our study may be explained by some critical points. Firstly, an early unclamping technique was used in almost all of the patients both to control active bleeders from tumor bed and to reduce WIT as much as possible. Another possible explanation could be the detailed work-up in the radiology unite on the 3-D CT before surgery. This method, although time-consuming, provides a better preoperative mapping of the renal vascular anatomy and makes it easier to understand tumor characteristics. The recent paper by Porpiglia et al. also addressed this issue. In that study, a 3D virtual model of the kidney was rendered from the CT-scans by the urologist and bioengineers, using medical software. According to the results of this study, patients who were preoperatively evaluated by this protocol were most likely to undergo RAPN successfully without warm ischemia of the healthy renal remnant ( 25 ). Lastly, we also believe that teamwork in the operating room including experienced bed-site physician and the scrub nurse plays an important role in order to improve the surgical outcomes of RAPN. Therefore, it can be concluded that if the surgical technique is standardized with correct preoperative planning, high trifecta rates may also be achieved even in the hands of low volume surgeons.

There are a few limitations of our study. Firstly, this is a single-center study by a single surgeon and secondly, this study is limited by its retrospective nature. Although patient information was registered in our database prospectively, retrospective analyzes may cause missed data which can affect results by reducing the size and power of the study. In addition, no control group was included in this study to compare the outcomes of the RAPN. If the study was designed as the comparative matched-paired analysis of low and high volume surgeons, there would have been a more concise conclusion for the results.

## CONCLUSIONS

RAPN is an accepted, safe and effective minimally invasive alternative in the treatment of renal masses. The present study suggests that reasonable trifecta rates can be achieved even by the low volume surgeons. However, match-paired and comparative studies are needed to confirm our findings.
